# In-depth proteomic profiling of the uveal melanoma secretome

**DOI:** 10.18632/oncotarget.10418

**Published:** 2016-07-06

**Authors:** Martina Angi, Helen Kalirai, Samuel Prendergast, Deborah Simpson, Dean E. Hammond, Michele C. Madigan, Robert J. Beynon, Sarah E. Coupland

**Affiliations:** ^1^ Liverpool Ocular Oncology Research Group, Department of Molecular and Clinical Cancer Medicine, Institute of Translational Medicine, University of Liverpool, Liverpool, UK; ^2^ Centre for Proteome Research, Institute of Integrative Biology, University of Liverpool, Liverpool, UK; ^3^ Department of Cellular and Molecular Physiology, Institute of Translational Medicine, University of Liverpool, Liverpool, UK; ^4^ School of Optometry, University of New South Wales, New South Wales, Australia; ^5^ Save Sight Institute, Ophthalmology, University of Sydney, New South Wales, Australia

**Keywords:** uveal melanoma, melanocytes, proteomics, secretome, exosome

## Abstract

Uveal melanoma (UM), the most common primary intraocular tumour in adults, is characterised by a high frequency of metastases to the liver, typically with a fatal outcome. Proteins secreted from cancer cells (‘secretome’) are biologically important molecules thought to contribute to tumour progression. We examined the UM secretome by applying a label-free nanoLCMS/MS proteomic approach to profile proteins secreted into culture media by primary UM tumours with a high− (HR; *n* = 11) or low− (LR; *n* = 4) metastatic risk, compared to normal choroidal melanocytes (NCM) from unaffected post-mortem eyes. Across the three groups, 1843 proteins were identified at a 1% false discovery rate; 758 of these by at least 3 unique peptides, and quantified. The majority (539/758, 71%) of proteins were classified as secreted either by classical (144, 19%), non-classical (43, 6%) or exosomal (352, 46%) mechanisms. Bioinformatic analyzes showed that the secretome composition reflects biological differences and similarities of the samples. Ingenuity^®^ pathway analysis of the secreted protein dataset identified abundant proteins involved in cell proliferation-, growth- and movement. Hepatic fibrosis/hepatic stellate cell activation and the mTORC1-S6K signalling axis were among the most differentially regulated biological processes in UM as compared with NCM. Further analysis of proteins upregulated ≥ 2 in HR-UM only, identified exosomal proteins involved in extracellular matrix remodelling and cancer cell migration/invasion; as well as classically secreted proteins, possibly representing novel biomarkers of metastatic disease. In conclusion, UM secretome analysis identifies novel proteins and pathways that may contribute to metastatic development at distant sites, particularly in the liver.

## INTRODUCTION

Uveal melanoma (UM), the most common primary intraocular cancer in adults, has an average incidence of 4 to 11 individuals per million per year worldwide [1, 2]. Despite successful treatment of the ocular tumor, about half of UM patients develop metastatic disease in the liver [3, 4]. Several clinical-, histopathological- and genetic features of primary UM are strongly associated with metastasis development [5–7]. In particular, loss of one copy of chromosome 3 (monosomy 3; M3) is strongly associated with the development of metastatic disease and a poor outcome [8, 9]. At present, however, effective treatment for metastatic UM remains limited (Reviewed in [10–13]). Moreover, the mechanisms behind the phenomenon of UM ‘hepatotropism’ are not well understood, and only a few research groups have been able to examine metastatic UM samples, which are difficult to access for various reasons [14–17].

Proteins that are secreted or shed from cells are termed the ‘secretome’, and represent an important biological subset of molecules with key roles in intercellular communication, cancer development and progression [18–20]. These secreted factors may be present in the bloodstream and could become valuable biomarkers of metastatic disease [21]. A pioneer study by Pardo *et al.* characterized the secretome of a panel of UM cell lines and one short-term primary UM culture by 2D DIGE and mass spectrometry (MS), identifying 133 proteins, a subset of which had also been reported in the secretome of other cancers [22]. Importantly, the authors recognised that the diversity of proteins identified in the short-term primary UM culture was much greater than those detected in UM cell lines, concluding that the former would be more informative for biologically relevant studies.

Using a liquid chromatography-tandem mass spectrometry (LC-MS/MS) label-free quantitative proteomics approach, we examined the proteins found in the secretomes from short-term cultures of primary UM cells stratified as “high” or “low” metastatic risk (HR or LR) according to their chromosome 3 status [23], and compared these with secretomes of normal choroidal melanocytes (NCM) from post-mortem human eyes. We hypothesised that the UM secretome is significantly altered compared with NCM and has the potential to elucidate key biological processes contributing to metastatic progression in this disease.

## RESULTS

### Primary cell cultures are representative of the original patient specimen

**Figure 1:**
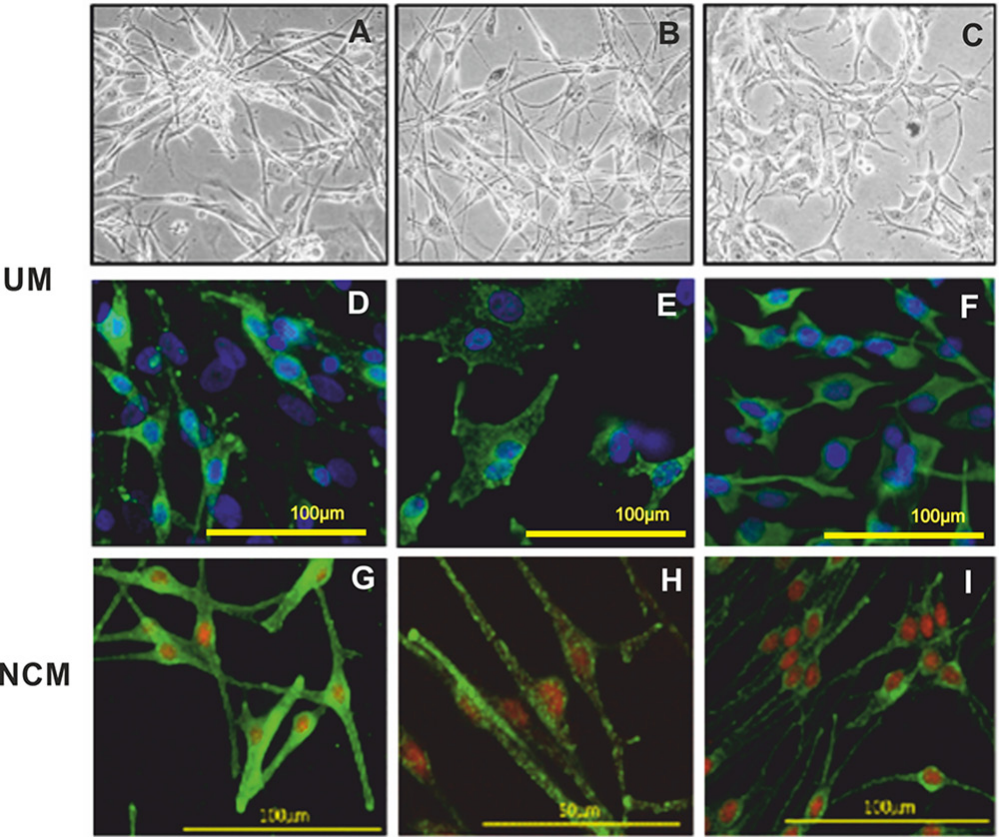
Representative images of UM and NCM cells in culture Brightfield images of UM cells with a spindle phenotype (**A** and **B**) and plump epithelioid-like cells (**C**). Immunofluorescence phenotyping of UM (**D, E** and **F**) and NCM (**G, H** and **I**) cells in culture. UM cells expressing (D) MelanA, (E) HMB45 and (F) vimentin. Positive staining was detected with Alexa 488 (green) and nuclei were counterstained with DAPI (blue). NCM expressing (G) MelanA, (H) gp100 and (I) HMB45. Positive staining was detected with Alexa 488 (green) and nuclei were counterstained with propidium iodide (red).

Between May and December 2012, fourteen UM cultures were fully characterized for morphological, immunohistochemical and genetic features, and used in the secretome analysis. The clinical data for these fourteen UM are provided in Supplementary Table 1. The chromosome 3 status of the original patient tumor and the short-term cultures of the primary UM cells was concordant in all cases; 10 cases were classified as HR (M3), and four as LR (Disomy 3; D3). The UM cells in culture grew either as a monolayer of spindle cells creating a neural-like network or as a layer of more epithelioid-like cells (Figure 1A–1C). Positive staining of > 60% of the cells in all cultures for MelanA, HMB45, vimentin and Microphthalmia-associated transcription factor (MITF) was also characteristic of their melanocytic origin (Figure 1D–1F).

Five NCM cultures were established from human post mortem eyes. The clinical details of the five donors are shown in Supplementary Table 2. NCM cells showed mainly spindle morphology, with evident pigmentation. They expressed the classical markers of the melanocytic lineage, i.e. MART-1/MelanA, HMB45 and gp100 as well as the proteins tyrosinase and TYRP1, which are specific to melanin synthesis (Figure 1G–1I).

### Identified proteins are qualitatively similar amongst samples and are largely secretory

**Figure 2:**
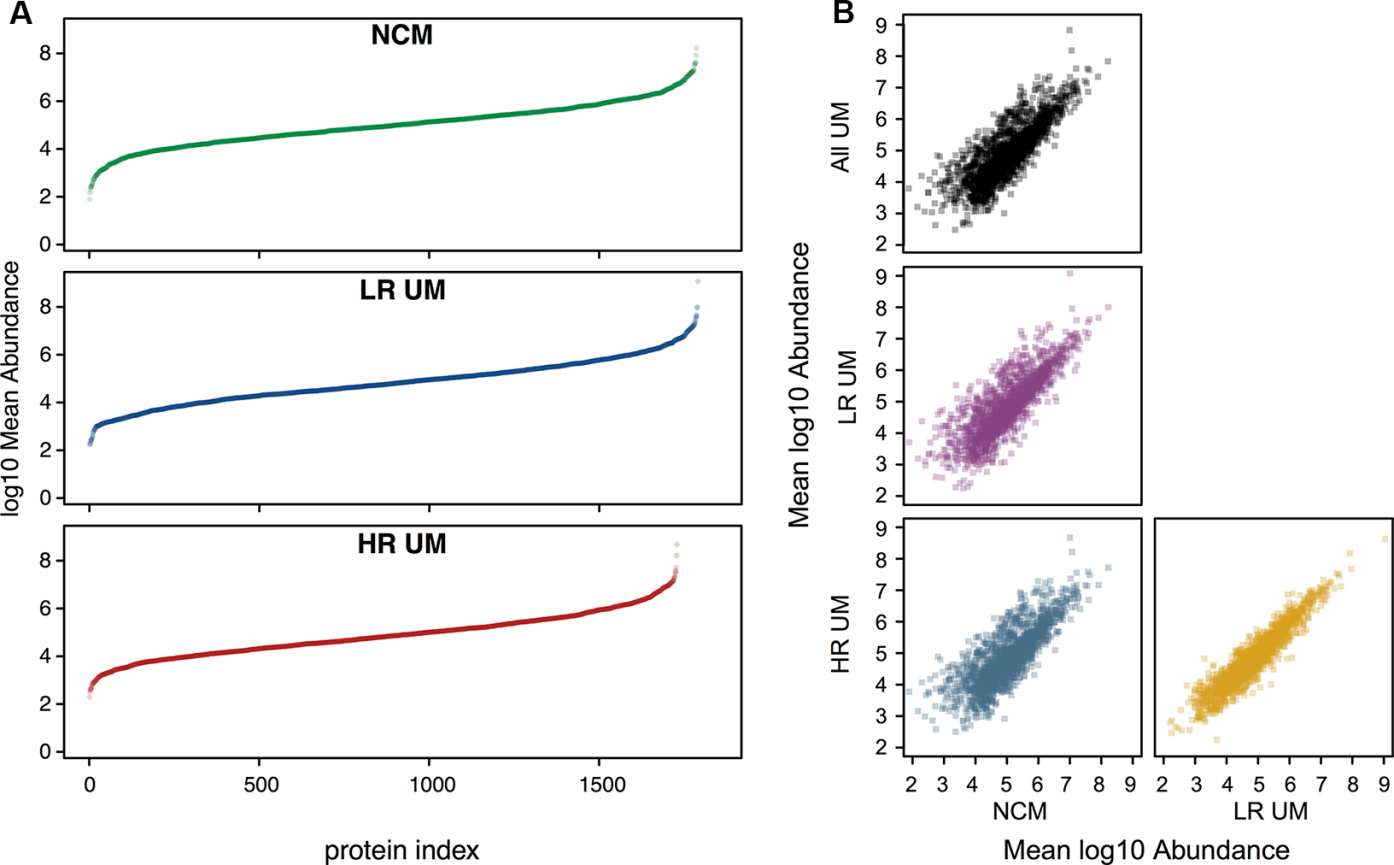
Proteomic profiles are qualitatively similar across all sample types Quantitative profiling of proteomes from different secretome samples. All samples were searched against the human UniProt reviewed database at an FDR of 1%. Where biological replicates were obtained, label free protein abundances were averaged prior to log transformation. (**A**) Mean abundance values were subsequently ranked and plotted in ascending order. In these plots, each point represents a single protein. All analyzes are scaled to the same axis limits for number of proteins and for abundance, to aid comparison between samples. (**B**) Pairwise comparisons of mean abundance between secretome groups. Also included is a comparison of mean NCM versus mean UM (top left panel).

Using standard proteomic workflows, we obtained protein profiles that were qualitatively similar across all three sample types (Supplementary Figures 1 and 2), consisting of a total of 1843 proteins (identified with a 1% False Discovery Rate (FDR)), and covering approximately 6 orders of magnitude of dynamic range (Figure 2).

To increase the confidence of downstream analyzes, only those proteins identified by 3 or more unique peptides were selected (758 of the 1843). Of these 758 proteins, 539 (71%) were classified as secreted either by classical (144, 19%), non-classical (43, 6%) or exosomal (352, 46%) mechanisms and defined as ‘secreted protein dataset’.

### Secretome composition reflects the biological differences and similarities of the samples

**Figure 3:**
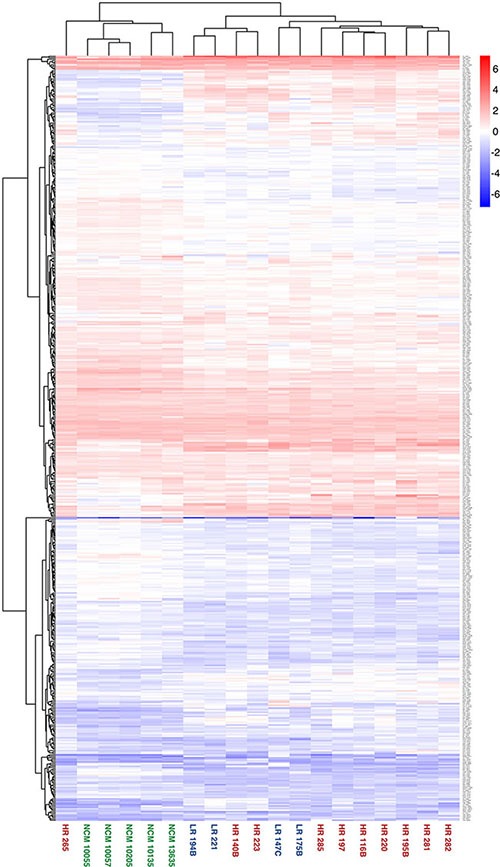
Heatmap showing highest level of separation discriminating between the secretomes from NCM and primary UM cells Hierarchical clustering of secretome sample based on label free abundance. The entire dataset of database matches for each secretome was used to direct a hierarchical clustering analysis, with log transformed label free quantification being used as the parameter. Samples from each subgroup are highlighted in a common colour; NCM = green, LR = blue, HR = red.

**Figure 4:**
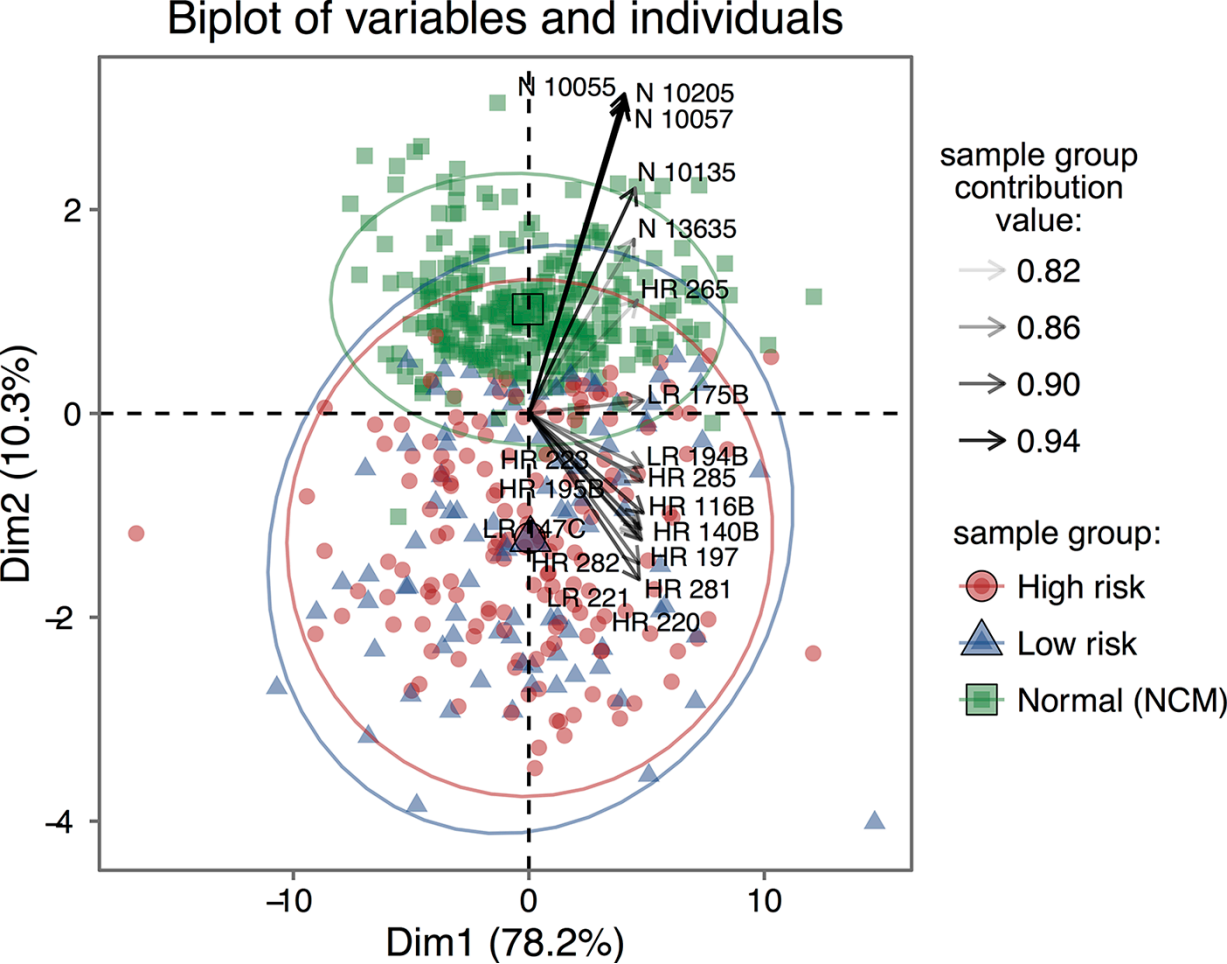
PCA biplot of individuals and variables showing a clear divergence between NCM and UM secretomes Principal components analysis of the label free quantitative data. Divergence in the projections of all components of the data are apparent, but that between NCM and UM subgroups is larger.

When the secreted protein dataset of 539 proteins was used to direct a hierarchical clustering analysis based on normalised log10-transformed protein abundance measurements, clear differences in the abundances of protein groups are apparent between secretome sample groups (Figure 3). This is also evident in the PCA biplot (Figure 4). In particular, a clear discrimination between NCM and UM secretomes was observed in both analyzes. A single outlier HR sample, HR265, was noted.

**Figure 5:**
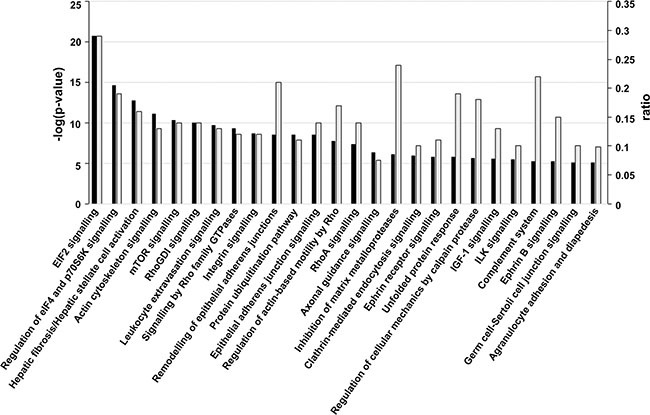
Canonical signalling pathways associated with UM Significant canonical signalling pathways (*p ≤ 0.00001;* Fisher's exact test) for proteins differentially expressed in UM versus NCM. The -log(*p*-value) (dark bars) and ratio (light grey bars) are plotted on the primary and secondary *Y*-axes, respectively. The ratio represents the number of detected molecules involved in the pathway/total number of known molecules in the pathway.

To identify enrichment terms associated with the 539 proteins, this dataset was uploaded to IPA^®^ with UM fold changes relative to NCM. The most highly-ranked biological process associated with the UM secretome was ‘cellular movement’ (Supplementary Table 3). A threshold *p*-value of ≤ 0.00001 (Fisher's exact test) was used to filter canonical signalling pathways associated with UM. Top canonical pathways included down-regulation of EIF2 signalling (Figure 5; Supplementary Table 4) as well as a significant involvement of proteins in hepatic fibrosis/hepatic stellate cell activation and translational control via the mTORC1-S6K signalling axis (Figure 5; Supplementary Table 4).

### Identification of proteins with biological importance in UM development and progression

The secreted protein dataset was further filtered to include only those proteins with a minimum fold change ≥ 2, *p* ≤ 0.05 (Mann Whitney U) in two-way comparisons between the individual datasets from HR, LR and NCM. This left a final set of 325 differentially expressed proteins across all three groups that were used for all subsequent analyzes, and are detailed in Supplementary Table 5.

To identify proteins of importance in UM, the differentially-expressed proteins discriminating HR-UM from NCM and LR-UM from NCM were compared. One hundred and sixty three proteins overlapped between the two datasets suggesting their general relevance to UM; 83 were up-regulated and 80 were down-regulated. Of interest, the majority of up-regulated proteins in this dataset (76%) were classically or non-classically secreted, whilst exosomal proteins represented the majority (85%) of those that were down-regulated.

Of potential significance to metastatic progression, a subset of 53 proteins differentially expressed in HR-UM only compared with both LR-UM and NCM were also identified (Supplementary Figure 3); 33 were up-regulated (Table 1) and 20 were down-regulated (Table 2). Thirteen up-regulated classically secreted proteins warrant further investigation as potential blood-borne biomarkers of metastatic risk (Table 1). Thirty-two exosomal proteins were identified in this dataset; 15 were up-regulated and 17 were down-regulated.

**Table 1:** Proteins up regulated in HR UM only

Accession	Unique peptides	Confidence score	Description	Secretory mechanism	Fold change HR vs LR
O15240	21	1487.97	Neurosecretory protein VGF GN = VGF	C	42.0
P48745	9	638.09	Protein V homolog GN = V	C	14.7
P35442	17	961.28	Thrombospondin-2 GN = THBS2	C	8.0
P16112	5	271.05	Aggrecan core protein GN = ACAN	C	6.0
O60462	7	343.53	Neuropilin-2 GN = NRP2	C	4.5
P19021	7	380.95	Peptidyl-glycine alpha-amidating monooxygenase GN = PAM	C	4.0
Q92743	7	442.47	Serine protease HTRA1 GN = HTRA1	C	3.0
P05121	7	470.84	Plasminogen activator inhibitor 1 GN = SERPINE1	C	2.9
P25391	13	648.55	Laminin subunit alpha-1 GN = LAMA1	C	2.7
P02462	5	405.57	Collagen alpha-1(IV) chain GN = COL4A1	C	2.5
P29279	10	494.08	Connective tissue growth factor GN = CTGF	C	2.5
Q16610	14	720.42	Extracellular matrix protein 1 GN = ECM1	C	2.4
Q16270	14	1280.19	Insulin-like growth factor-binding protein 7 GN = IGFBP7	C	2.4
P29120	7	420.62	Neuroendocrine convertase 1 GN = PCSK1	NC	16.2
P98155	4	166.49	Very low-density lipoprotein receptor GN = VLDLR	NC	6.0
Q9UBG0	5	213.11	C-type manse receptor 2 GN = MRC2	NC	4.2
Q9Y639	3	146.05	Neuroplastin GN = NPTN	NC	2.3
P05067	12	675.22	Amyloid beta A4 protein GN = APP	NC	2.1
P00736	5	344.66	Complement C1r subcomponent GN = C1R	E	9.0
P09871	10	600.05	Complement C1s subcomponent GN = C1S	E	6.1
O00161	3	118.77	Synaptosomal-associated protein 23 GN = SNAP23	E	4.8
Q06481	17	976.38	Amyloid-like protein 2 GN = APLP2	E	3.9
P34896	3	144.82	Serine hydroxymethyltransferase, cytosolic GN = SHMT1	E	3.9
O75955	4	315.33	Flotillin-1 GN = FLOT1	E	3.8
Q9NZM1	22	1209.91	Myoferlin GN = MYOF	E	3.3
Q86UX7	4	217.02	Fermitin family homolog 3 GN = FERMT3	E	3.3
P08582	7	342.22	Melanotransferrin GN = MFI2	E	2.9
P16070	5	361.46	CD44 antigen GN = CD44	E	2.6
O60568	8	446.68	Procollagen-lysine,2-oxoglutarate 5-dioxygenase 3 GN = PLOD3	E	2.3
O00159	8	409.62	Unconventional myosin-Ic GN = MYO1C	E	2.2
P11717	22	1134.74	Cation-independent mannose-6-phosphate receptor GN = IGF2R	E	2.2
P06756	4	185.83	Integrin alpha-V GN = ITGAV	E	2.1
Q14697	6	400.91	Neutral alpha-glucosidase AB GN = GANAB	E	3.4

C = Classical secretion; NC = Non-classical secretion; E = exosomal.

**Table 2:** Proteins down regulated in HR UM only

Accession	Unique peptides	Confidence score	Description	Secretory mechanism	Fold change LR vs HR
P10451	5	261.11	Osteopontin GN = SPP1	C	8.1
Q9Y2W1	3	227.35	Thyroid hormone receptor-associated protein 3 GN = THRAP3	E	7.0
Q16555	11	936.62	Dihydropyrimidinase-related protein 2 GN = DPYSL2	E	4.9
P16401	8	515.65	Histone H1.5 GN = HIST1H1B	E	3.3
P09651	7	742.34	Heterogeneous nuclear ribonucleoprotein A1 GN = HNRNPA1	E	3.2
P07910	9	451.12	Heterogeneous nuclear ribonucleoproteins C1/C2 GN = HNRNPC	E	3.2
Q9UBX1	3	179.26	Cathepsin F GN = CTSF	E	3.0
P16949	3	259.14	Stathmin GN = STMN1	E	3.0
Q14980	10	445.09	Nuclear mitotic apparatus protein 1 GN = NUMA1	E	2.5
P53999	3	165.03	Activated RNA polymerase II transcriptional coactivator p15 GN = SUB1	E	2.5
P13861	5	195.4	cAMP-dependent protein kinase type II-alpha regulatory subunit GN = PRKAR2A	E	2.4
Q71DI3	5	414.24	Histone H3.2 GN = HIST2H3A	E	2.4
P14866	8	403.33	Heterogeneous nuclear ribonucleoprotein L GN = HNRNPL	E	2.4
O60869	3	142.89	Endothelial differentiation-related factor 1 GN = EDF1	E	2.3
P62805	11	966.97	Histone H4 GN = HIST1H4A	E	2.2
Q9Y265	4	222.71	RuvB-like 1 GN = RUVBL1	E	2.1
P09211	4	269.11	Glutathione S-transferase P GN = GSTP1	E	2.1
P29401	14	892.15	Transketolase GN = TKT	E	2.1
P13667	7	487.96	Protein disulfide-isomerase A4 GN = PDIA4	NC	2.6
Q93052	3	107.53	Lipoma-preferred partner GN = LPP	NC	2.4

C = Classical secretion; NC = Non-classical secretion; E = exosomal.

## DISCUSSION

To our knowledge, this study describes the largest and most comprehensive characterisation of UM secreted proteomes to date; 1843 proteins were identified in all sample subsets at a 1% FDR cut-off, and 758 of these with at least 3 unique peptides. Moreover, we analyze for the first time the secretome from a panel of primary UM cells stratified as LR or HR according to their chromosome 3 status, and NCM in short-term culture, with the aim of recapitulating the multiform biological landscape of UM. Importantly, our data reveal that primary cultured UM cells can be discriminated from NCM based on their secreted protein profile and that differential protein secretion was evident between the LR and the HR-UM. Of note was a single HR UM sample, HR265, which failed to cluster with other UM samples. Although the reason for this is not clinically apparent, it may be explained by the fact that this culture was the only one to be passaged.

Cell lines have been widely used for secretome studies because they are easily accessible, grow well in culture, and can be used for multiple experiments with minimal inter-sample variability. This homogeneity, however, is also a major limitation, as it does not reflect the biological variability seen in patients. In this study, we used short-term primary UM cultures that, although difficult to obtain, more closely represent tumors *in vivo*, as evidenced by our phenotypic and genetic comparison of the primary cells with the original patient tumor. One disadvantage of using primary UM samples, however, was the paucity of cultures established from LR-D3 UM. This may be due to the fact that the surgical samples used for culture were often from large tumors, which is an established parameter of UM with a higher metastatic risk [33]. The identification of truly secreted proteins can often be complicated by cell leakage; in particular an absence of serum in the culture medium can cause increased cell death. We performed preliminary analyzes, as recently described by Villareal and colleagues [34], to determine the contribution of decreased cell viability and apoptosis to the secretome composition. We found, at 48 hours, that cell viability was ≥ 95% and evidence of cell death was not a prominent feature (data not shown).

Another unique aspect of this work is the production of secretome from primary NCM enabling the identification of secreted proteins that were significantly altered specifically in the UM cells. Human NCM are difficult to obtain and they are difficult to successfully propagate in culture [35]. To the best of our knowledge, this is the first secretome study performed in such cells.

A variety of proteomic techniques are described in the analysis of cancer secretomes from cell lines or primary cultures, commonly identifying up to several hundred proteins in the conditioned medium. Indeed, analysis of the secretome from a single primary UM culture using 2D gel electrophoresis and mass spectrometry identified 133 proteins, approximately 32% of which were predicted to be secreted [22]. Using novel proteomic methods that reduced sample handling, avoided enrichment steps such as ultrafiltration, and extracted proteins from the dilute sample using StrataClean beads followed by on-bead digestion, we were able to identify a much larger cohort of proteins across the UM and NCM cultures; 1843 proteins at a 1% FDR, reducing to 785 proteins identified with at least 3 unique peptides. Interestingly, 112 proteins (84%) of the previously-reported dataset [22] were also present within our cohort. We also compared our secreted protein dataset with proteins reported in two studies that examined differentially expressed proteins in UM tissue from patients who went on to develop metastases and those who did not [36, 37]. In the study by Linge et al. eight of the 14 proteins reported as differentially expressed between primary UM that metastasised and those that did not, were found in our secreted protein dataset; PDIA3, VIM/HEXA, SELENBP1, ERP29, TPI1, PARK7, EIF2S1 and RPSA [36]. None were significantly differentially expressed between HR and LR UM secretomes (Mann Whitney U; *p* > 0.05). Crabb et al. identified 30 proteins as significantly elevated and 28 proteins as significantly decreased only in primary UM from patients who went on to develop metastasis [37]. Twenty of the 58 differentially expressed proteins were found in our secreted protein dataset; of these only, WARS and PMEL, were similarly elevated in HR vs LR UM and MUC18 was similarly decreased in HR vs LR UM, although these differences were not statistically significant (Mann Whitney U; *p* > 0.05).

We further demonstrate that the majority (71%) of identified proteins are predicted to be secreted; 19% as classically and 6% as non-classically secreted, and 46% contained within exosomes. Exosomes are microvesicles secreted by most cell types that have recently received a huge amount of interest as a mechanism by which protein cargo can be transported from one site to another as an important means of cell-cell communication (Reviewed in [38, 39]).

Bioinformatic analysis of the data identified proteins and molecular pathways significantly altered in UM as compared with NCM, and those significantly altered in HR- UM compared with LR- UM and NCM. Indeed, several of the hallmarks of cancer were activated in UM as compared with NCM, in particular cell migration and invasion, and activation and trafficking of immune cells. Pathway analysis also suggested that hepatic fibrosis/hepatic stellate cell activation was amongst the most differentially regulated biological process in UM as compared with NCM. Hepatic stellate cell activation is associated with increased tumor growth and invasion, angiogenesis and suppression of the anti-tumor immune response in other cancers [40], and is of particular interest for UM metastases, which are located predominantly in the liver. Moreover, there is a recently described role of exosomes in initiation of the pre-metastatic niche [41, 42]. Interestingly, the putative exosomal proteins highly upregulated in HR-UM only (Table 1), have roles in extracellular matrix (ECM) remodelling and facilitating cancer cell migration and invasion [43]. In particular, C1s/C1r are reported to degrade collagen and are involved in activation of CUB domain containing proteins that regulate many aspects of cancer progression and metastasis [44]. SNAP23 is a key regulator of the membrane-membrane fusion events required for intracellular membrane traffic and has been reported to traffic matrix metalloproteases during degradation of ECM substrates, thus enabling cellular invasion [45], whilst myoferlin is involved in increased tumor associated angiogenesis [46, 47]. The differential expression of exosomal proteins both between HR and LR UM, and also between all UM and NCM may suggest basic differences in exosome biogenesis and trafficking. Further investigation of exosomal proteins and their function in UM is warranted.

Increasing interest has developed in the cancer field in serum biomarkers that may represent a minimally invasive method for detection of metastatic disease, and the secretome is a rich source of potential biomarkers for this purpose. In UM, a number of proteins have already been examined in patient sera including OPN [48], MIA [49], S100ß [50], GDF15 [51], PARK7 [52], ME20 [53], soluble c-Met [54] and IGF [55], each demonstrating varying degrees of sensitivity and specificity in the identification of patients with disseminated disease compared with metastasis-free UM patients and/or normal healthy controls. Of these proteins OPN, MIA, GDF15, PARK7 and ME20 were detected in the current study; however, only MIA and GDF15 demonstrated significantly higher levels in the secretome of UM compared with NCM, with no significant difference between the levels detected in HR- and LR-UM. An additional 13 classically-secreted proteins, which are significantly elevated in the HR-UM as compared with either the LR-UM or the NCM, are presented in this study that warrant further validation in patient blood specimens. Of note, circulating levels of IGFBP7 and THBS2 have been associated with outcome in high-grade soft tissue sarcoma and non-small cell lung cancer, respectively [56, 57]. It should be emphasised, however, that many of the proteins described above are also elevated in the serum in other conditions.

In summary, we established short-term cultures from UM and NCM for secretome production. Using label-free MS, we identified and quantified a large number of proteins, and demonstrated that the vast majority of these were secreted, either by classical, ‘non-classical’, or exosomal mechanisms. Bioinformatic analyzes showed that the secretome composition reflects the biological differences and similarities of the samples. Following assessment of the UM secretome for biological function, pathway analysis and interaction networks, the biological process that was highlighted was ‘cellular movement’, whilst one of the top-featured canonical pathways involved ‘hepatic fibrosis’ and ‘hepatic stellate cell activation’. These data provide further insight into the UM metastatic process, and highlight potential biomarkers of metastatic disease in UM patients.

## MATERIALS AND METHODS

### Establishment and characterisation of primary UM cell cultures

Following local and national research ethics committee approvals (HRA REC Ref 11/NW/0568), fresh primary tumor specimens of consenting UM patients undergoing surgical removal as treatment were obtained from the Liverpool Ocular Oncology Biobank (HRA REC Ref 11/NW/0249).

For each UM sample, a single cell suspension was obtained by mincing the tissue, followed by incubation with 500 U/mL of type I collagenase (Sigma-Aldrich Company Ltd, Dorset, UK) at 37°C for approximately 1 hr, with occasional agitation of the solution. Single cells were recovered by centrifugation (250 × g for 2 min) and re-suspended in primary culture medium (1:1 αMEM (Life Technologies Ltd, Paisley, UK): Quantum 3-21, (PAA Laboratories Ltd, UK), 10% fetal bovine serum, plus antibiotics and 2 mM L-Glutamine). The cells were seeded into two 75 cm^2^ tissue culture flasks (Falcon, VWR international, Leicestershire, UK) each at a density of 1.5 × 10^6^ cells, and two 8-well chamber slides (Fisher Scientific UK Ltd, Loughborough, UK) at a density of 50,000 cells/well in primary culture medium. The cells were grown to approximately 75–80% confluence.

The following characteristics of each primary UM cell culture was recorded and used to determine its similarity with the original patient tumor:

1. Cell morphology documented by digital images.
2. Expression of melanoma markers; cells in chamber slides were fixed with 3.7% formaldehyde in phosphate buffered saline (PBS) for 10min. Immunofluorescence staining was performed as previously described [24], using the following antibodies for 30min at room temperature (RT); mouse anti-human HMB45 (DAKO, Ely, Cambridgeshire, UK; 1:200), mouse anti-human MelanA (DAKO; 1:100), mouse anti-human vimentin (DAKO; 1:200), mouse anti-human MITF (Novocastra, Newcastle, UK; 1:100) and mouse anti-human αSMA (DAKO 1:600).
3. Chromosomal alterations; following collection of the secretome, primary UM cells were harvested and lysed in 180μL of ATL buffer (Qiagen Ltd, Manchester, UK) and stored at −20°C for DNA extraction. DNA was extracted and quantified as previously described [25]. Depending on the DNA concentration obtained, either microsatellite analysis (MSA) of chromosome 3 or multiplex ligation dependent probe amplification (MLPA) of chromosomes 1, 3, 6 and 8 was performed, and compared to the genetic profile of the original UM specimen. As previously described, tumors were stratified as HR if they showed M3, or LR if they were found to have a normal chromosome 3 copy number (disomy 3; D3) [23, 25, 26].

### Establishment of human choroidal melanocyte cultures

NCM were isolated from consented human post-mortem eyes with a delay of less than 18 hours, from the Lions New South Wales Eye Bank. The study was performed with approval from the University of Sydney and University of New South Wales Human Research Ethics Committee. NCM were isolated and grown in melanocyte growth medium as previously described [27]. Passage 1–3 melanocytes were used for all experiments. Immunofluorescence staining was performed on a subset of each NCM culture grown in 8-well chamber slides. In brief, cells in chamber slides were fixed in 2% paraformaldehyde in PBS (pH 7.4) for 20 min at RT, rinsed in PBS and blocked in 5% BSA. Primary antibodies (all from Thermo Fisher Scientific Australia Pty Ltd, Scoresby Vic) used to assess the NCM included: Mel Ab-3 (HMB45+HMB50); Tyrosinase (T311); tyrosinase-related protein 1, TRYP1 (TA99); MART-1 Ab-3; gp-100; and vimentin. Cells were incubated with primary antibodies overnight at 4°C, followed by incubation with the appropriate species-specific Alexa-488 conjugated secondary antibody (1:1000, Molecular Probes, USA) for 1 hr at RT. Nuclei were counterstained with either DAPI or propidium iodide, prior to cover-slipping.

### Secretome sample preparation and collection

Using a protocol originally developed for glioblastoma cells [28], UM cell monolayers were rinsed three times with 10 mL of PBS, incubated with 10mL of serum free medium (SFM; phenol-red free αMEM, Life Technologies Ltd) for 1 hr, and rinsed once again with fresh SFM. Cells were then incubated with 8 mL of SFM for 48 hr, and the conditioned medium at the end of the incubation period was defined as the secretome. For each sample, the secretome from both 75 cm^2^ flasks was pooled into a 30 ml universal tube, and centrifuged at 300 × g for 5mins at 4°C to pellet any floating cells. These were pooled with cells detached from the bottom of the flask and viability was determined by trypan blue dye exclusion to be ≥ 95%.

The supernatant was aliquoted and stored at −80°C until further analysis. The cell pellet was used for chromosomal analysis as described above.

The same protocol was adopted for secretome production from NCM in culture, but with a final volume of secretome for each NCM sample of 1.4 mL.

### Proteomic analysis of secretome samples

All proteomic analyzes were conducted only once the prospective collection of all secretome samples had been completed. The total secretome protein content of each UM sample was determined using a standard Bradford assay. In order to concentrate these dilute protein solutions, we made use of absorption onto StrataClean™ beads (Stratagene^®^, Hycor Biomedical Ltd., Edinburgh, UK), an established way of concentrating protein prior to proteome analysis [29]. For on-bead digestion the beads were re-suspended in 80 μL of 25 mM ambic and 5 μL of 1%(w/v) Rapigest (Waters, Hertfordshire, UK) in 25 mM ambic, and the samples heated at 80°C for 10 min. Samples were then reduced, by the addition of 5 μL of 60 mM DTT and heated at 60°C for 10 min, before being cooled prior to addition of 5 μL of 180 mM iodoacetamide and incubation at RT for 30 min in the dark. Porcine trypsin (sequencing grade, Sigma) (1 μg) was added and the sample was incubated at 37°C overnight on a rotary mixer. Peptide digests were subsequently acidified by the addition of 1 μL of trifluoroacetic acid (TFA) and incubated at 37°C for 45 min. Following centrifugation at 17,000 × g for 30 min, the clarified supernatants were transferred to 0.5 mL low-bind tubes and further centrifuged (17,000 × g for 30 min). 10 μL of each peptide mixture was prepared for nano LC-MS/MS.

Digests (2 μL) from each sample were loaded onto a trap column (Acclaim PepMap 100, 2 cm × 75 μm inner diameter, C_18_, 3 μm, 100 Å) at 5 μl min^−1^ with an aqueous solution containing 0.1%(v/v) TFA and 2%(v/v) acetonitrile. After 3 min, the trap column was set in-line with an analytical column (Easy-Spray PepMap^®^ RSLC 50 cm × 75 μm inner diameter, C_18_, 2 μm, 100 Å) (Dionex). Peptides were loaded in 0.1%(v/v) formic acid and eluted with a linear gradient of 3.8 – 40% buffer B (HPLC grade acetonitrile 80%(v/v) with 0.1%(v/v) formic acid) over 95 min at 300 nl min^−1^, followed by a washing step (5 min at 99% solvent B) and an equilibration step (15 min at 3.8% solvent). All peptide separations were carried out using an Ultimate 3000 nano system (Dionex/Thermo Fisher Scientific). The column was operated at a constant temperature of 35°C and the LC system coupled to a Q-Exactive mass spectrometer (Thermo Fisher Scientific). The Q-Exactive was operated in data-dependent mode with survey scans acquired at a resolution of 70,000 at m/z 200. Up to the top 10 most abundant isotope patterns with charge states +2, +3 and/or +4 from the survey scan were selected with an isolation window of 2.0 Th for fragmentation by higher energy collisional dissociation with normalized collision energies of 30. The maximum ion injection times for the survey scan and the MS/MS scans were 250 and 100 ms, respectively, and the ion target value was set to 1E6 for survey scans and 1E5 for the MS/MS scans. Repetitive sequencing of peptides was minimized through dynamic exclusion of the sequenced peptides for 20 sec [30].

### Protein identification and quantification

Raw mass spectral data files were processed using Progenesis-QI (v2; Nonlinear Dynamics) to determine total protein abundances. All raw files were initially automatically aligned, according to retention time, to produce an aggregate LC-MS map, from which peptide feature charge-states +1 and > +7 were excluded. This aggregated spectral map contains MS features from all aligned runs enabling maximal protein identification across all samples. Data were then separated into three experimental sample groups; (1) 5 NCM samples, (2) 4 LR UM samples, and (3) 10 HR UM samples. An aggregate peak list file (.mgf format) containing only MS data relating to peptides ranked 1 – 5, was then searched against a human reviewed UniProt database (date: 4/29/2015) using the Mascot search engine (version 2.4.1; Matrix Science, UK). A precursor ion tolerance of 10ppm and a fragment ion tolerance of 0.01Da were used, with carbamidomethylation of cysteine set as a fixed modification and oxidation of methionine as a variable modification. Trypsin was the specified enzyme and one missed cleavage was allowed.

Protein quantification was based on averaging the individual abundances for every unique peptide for each protein and comparing them relatively across sample runs and between sample groups (NCM, LR and HR).

### Identification of secreted proteins

Stringent criteria were applied to include only proteins identified with a FDR of < 1% and by at least three unique peptides. Secreted proteins were defined using a combination of SignalP v4.1 [31] and SecretomeP v2.0 [32] prediction methods, and UniProt keyword annotations e.g. extracellular space and exosome. Signal P and Secretome P are computational methods based on machine learning algorithms. SignalP predicts N-terminal signal peptides, whereas SecretomeP predicts secretory proteins following non-classical, signal peptide-independent mechanisms. The default NN-score and D-score cut-offs were used for SecretomeP and SignalP respectively. Other routes of non-classical secretion were examined using keyword annotations in Uniprot of ‘extracellular space’ and ‘exosome’. Potential exosomal proteins were also identified by converting the Entrez Gene IDs in the ExoCarta download 5 (release date 29th July 2015) into protein accession numbers for cross-referencing.

Where more than one classification could be assigned to a protein the predictions were weighted as follows; (1) classically secreted proteins (SignalP), (2) non-classically secreted proteins (SecretomeP), (3) exosomal proteins (Exocarta and Uniprot), and (4) non-secreted. Only proteins classified into groups (1) – (3) were used in downstream analyzes.

### Statistics and bioinformatics analyzes

Data was visualised and statistics performed using various R (v3.2.3) packages and ggplot2 (v2.1.0). ANOVA and Mann-Whitney U statistical methods were used to identify significantly (*p* ≤ 0.05) differentially expressed proteins. Data were uploaded together with fold changes into the Ingenuity^®^ Pathway Analysis (IPA) software (Ingenuity Systems, MountainView, CA) to investigate molecular and biological functions of the differentially expressed proteins.

## SUPPLEMENTARY MATERIALS FIGURES AND TABLES









## References

[1] Singh AD, Topham A (2003). Incidence of uveal melanoma in the United States: 1973–1997. Ophthalmology.

[2] Yonekawa Y, Kim IK (2012). Epidemiology and management of uveal melanoma. Hematol Oncol Clin North Am.

[3] Damato B (2010). Does ocular treatment of uveal melanoma influence survival?. Br J Cancer.

[4] Diener-West M, Reynolds SM, Agugliaro DJ, Caldwell R, Cumming K, Earle JD, Hawkins BS, Hayman JA, Jaiyesimi I, Jampol LM, Kirkwood JM, Koh WJ, Robertson DM (2005). Development of metastatic disease after enrollment in the COMS trials for treatment of choroidal melanoma: Collaborative Ocular Melanoma Study Group Report No. 26. Arch Ophthalmol.

[5] Field MG, Harbour JW (2014). Recent developments in prognostic and predictive testing in uveal melanoma. Curr Opin Ophthalmol.

[6] Kivela T, Kujala E (2013). Prognostication in eye cancer: the latest tumor, node, metastasis classification and beyond. Eye (Lond).

[7] Coupland SE, Lake SL, Zeschnigk M, Damato BE (2013). Molecular pathology of uveal melanoma. Eye (Lond).

[8] Prescher G, Bornfeld N, Hirche H, Horsthemke B, Jockel KH, Becher R (1996). Prognostic implications of monosomy 3 in uveal melanoma. Lancet.

[9] Damato B, Duke C, Coupland SE, Hiscott P, Smith PA, Campbell I, Douglas A, Howard P (2007). Cytogenetics of uveal melanoma: a 7-year clinical experience. Ophthalmology.

[10] Nabil AA, Marie S, Marc-Henri S, Nathalie C, Laurence D, Sophie PN, Olivier L, Sergio RR (2015). Upcoming translational challenges for uveal melanoma. Br J Cancer.

[11] Luke JJ, Triozzi PL, McKenna KC, Van Meir EG, Gershenwald JE, Bastian BC, Gutkind JS, Bowcock AM, Streicher HZ, Patel PM, Sato T, Sossman JA, Sznol M (2015). Biology of advanced uveal melanoma and next steps for clinical therapeutics. Pigment Cell Melanoma Res.

[12] Pereira PR, Odashiro AN, Lim LA, Miyamoto C, Blanco PL, Odashiro M, Maloney S, De Souza DF, Burnier MN (2013). Current and emerging treatment options for uveal melanoma. Clin Ophthalmol.

[13] Mariani P, Servois V, Piperno-Neumann S (2012). Therapeutic options in metastatic uveal melanoma. Dev Ophthalmol.

[14] McCarthy C, Kalirai H, Lake SL, Dodson A, Damato BE, Coupland SE (2016). Insights into genetic alterations of liver metastases from uveal melanoma. Pigment Cell Melanoma Res.

[15] Luscan A, Just PA, Briand A, Burin des Roziers C, Goussard P, Nitschke P, Vidaud M, Avril MF, Terris B, Pasmant E (2015). Uveal melanoma hepatic metastases mutation spectrum analysis using targeted next-generation sequencing of 400 cancer genes. Br J Ophthalmol.

[16] Trolet J, Hupe P, Huon I, Lebigot I, Decraene C, Delattre O, Sastre-Garau X, Saule S, Thiery JP, Plancher C, Asselain B, Desjardins L, Mariani P (2009). Genomic profiling and identification of high-risk uveal melanoma by array CGH analysis of primary tumors, liver metastases. Invest Ophthalmol Vis Sci.

[17] Meir T, Dror R, Yu X, Qian J, Simon I, Pe'er J, Chowers I (2007). Molecular characteristics of liver metastases from uveal melanoma. Invest Ophthalmol Vis Sci.

[18] Lin Q, Tan HT, Lim HS, Chung MC (2013). Sieving through the cancer secretome. Biochim Biophys Acta.

[19] Paltridge JL, Belle L, Khew-Goodall Y (2013). The secretome in cancer progression. Biochim Biophys Acta.

[20] Caccia D, Zanetti Domingues L, Micciche F, De Bortoli M, Carniti C, Mondellini P, Bongarzone I (2011). Secretome compartment is a valuable source of biomarkers for cancer-relevant pathways. J Proteome Res.

[21] Schaaij-Visser TB, de Wit M, Lam SW, Jimenez CR (2013). The cancer secretome, current status and opportunities in the lung, breast and colorectal cancer context. Biochim Biophys Acta.

[22] Pardo M, Garcia A, Antrobus R, Blanco MJ, Dwek RA, Zitzmann N (2007). Biomarker discovery from uveal melanoma secretomes: identification of gp100 and cathepsin D in patient serum. J Proteome Res.

[23] Caines R, Eleuteri A, Kalirai H, Fisher AC, Heimann H, Damato BE, Coupland SE, Taktak AF (2015). Cluster analysis of multiplex ligation-dependent probe amplification data in choroidal melanoma. Mol Vis.

[24] Kalirai H, Damato BE, Coupland SE (2011). Uveal melanoma cell lines contain stem-like cells that self-renew, produce differentiated progeny, and survive chemotherapy. Invest Ophthalmol Vis Sci.

[25] Lake SL, Kalirai H, Dopierala J, Damato BE, Coupland SE (2012). Comparison of formalin-fixed and snap-frozen samples analyzed by multiplex ligation-dependent probe amplification for prognostic testing in uveal melanoma. Invest Ophthalmol Vis Sci.

[26] Coupland SE, Kalirai H, Ho V, Thornton S, Damato BE, Heimann H (2015). Concordant chromosome 3 results in paired choroidal melanoma biopsies and subsequent tumour resection specimens. Br J Ophthalmol.

[27] Lai K, Di Girolamo N, Conway RM, Jager MJ, Madigan MC (2007). The effect of ultraviolet radiation on choroidal melanocytes and melanoma cell lines: cell survival and matrix metalloproteinase production. Graefes Arch Clin Exp Ophthalmol.

[28] Polisetty RV, Gupta MK, Nair SC, Ramamoorthy K, Tiwary S, Shiras A, Chandak GR, Sirdeshmukh R (2011). Glioblastoma cell secretome: analysis of three glioblastoma cell lines reveal 148 non-redundant proteins. J Proteomics.

[29] McLean L, Hurst JL, Gaskell CJ, Lewis JC, Beynon RJ (2007). Characterization of cauxin in the urine of domestic and big cats. J Chem Ecol.

[30] Hammond DE, Claydon AJ, Simpson DM, Edward D, Stockley P, Hurst JL, Beynon RJ (2016). Proteome Dynamics: Tissue Variation in the Kinetics of Proteostasis in Intact Animals. Mol Cell Proteomics.

[31] Petersen TN, Brunak S, von Heijne G, Nielsen H (2011). SignalP 4. 0: discriminating signal peptides from transmembrane regions. Nat Methods.

[32] Bendtsen JD, Jensen LJ, Blom N, Von Heijne G, Brunak S (2004). Feature-based prediction of non-classical and leaderless protein secretion. Protein Eng Des Sel.

[33] Seddon JM, Albert DM, Lavin PT, Robinson N (1983). A prognostic factor study of disease-free interval and survival following enucleation for uveal melanoma. Arch Ophthalmol.

[34] Villarreal L, Méndez O, Salvans C, Gregori J, Baselga J, Villanueva J (2013). Unconventional secretion is a major contributor of cancer cell line secretomes. Mol Cell Proteomics.

[35] Pardo M, Dwek RA, Zitzmann N (2007). Proteomics in uveal melanoma research: opportunities and challenges in biomarker discovery. Expert Rev Proteomics.

[36] Linge A, Kennedy S, O'Flynn D, Beatty S, Moriarty P, Henry M, Clynes M, Larkin A, Meleady P (2012). Differential expression of fourteen proteins between uveal melanoma from patients who subsequently developed distant metastases versus those who did Not. Invest Ophthalmol Vis Sci.

[37] Crabb JW, Hu B, Crabb JS, Triozzi P, Saunthararajah Y, Tubbs R, Singh AD (2015). iTRAQ Quantitative Proteomic Comparison of Metastatic and Non-Metastatic Uveal Melanoma Tumors. PLoS One.

[38] Kalluri R (2016). The biology and function of exosomes in cancer. J Clin Invest.

[39] Pitt JM, Kroemer G, Zitvogel L (2016). Extracellular vesicles: masters of intercellular communication and potential clinical interventions. J Clin Invest.

[40] Kang N, Gores GJ, Shah VH (2011). Hepatic stellate cells: partners in crime for liver metastases?. Hepatology.

[41] Peinado H, Aleckovic M, Lavotshkin S, Matei I, Costa-Silva B, Moreno-Bueno G, Hergueta-Redondo M, Williams C, Garcia-Santos G, Ghajar C, Nitadori-Hoshino A, Hoffman C, Badal K (2012). Melanoma exosomes educate bone marrow progenitor cells toward a pro-metastatic phenotype through MET. Nat Med.

[42] Costa-Silva B, Aiello NM, Ocean AJ, Singh S, Zhang H, Thakur BK, Becker A, Hoshino A, Mark MT, Molina H, Xiang J, Zhang T, Theilen TM (2015). Pancreatic cancer exosomes initiate pre-metastatic niche formation in the liver. Nat Cell Biol.

[43] Gilkes DM, Semenza GL, Wirtz D (2014). Hypoxia and the extracellular matrix: drivers of tumour metastasis. Nat Rev Cancer.

[44] Yamaguchi K, Sakiyama H, Matsumoto M, Moriya H, Sakiyama S (1990). Degradation of type I and II collagen by human activated C1-s. FEBS Lett.

[45] Kean MJ, Williams KC, Skalski M, Myers D, Burtnik A, Foster D, Coppolino MG (2009). VAMP3, syntaxin-13 and SNAP23 are involved in secretion of matrix metalloproteinases, degradation of the extracellular matrix and cell invasion. J Cell Sci.

[46] Fahmy K, Gonzalez A, Arafa M, Peixoto P, Bellahcene A, Turtoi A, Delvenne P, Thiry M, Castronovo V, Peulen O (2016). Myoferlin plays a key role in VEGFA secretion and impacts tumor-associated angiogenesis in human pancreas cancer. Int J Cancer.

[47] Yu C, Sharma A, Trane A, Utokaparch S, Leung C, Bernatchez P (2011). Myoferlin gene silencing decreases Tie-2 expression *in vitro* and angiogenesis *in vivo*. Vascul Pharmacol.

[48] Kadkol SS, Lin AY, Barak V, Kalickman I, Leach L, Valyi-Nagy K, Majumdar D, Setty S, Maniotis AJ, Folberg R, Pe'er J (2006). Osteopontin expression and serum levels in metastatic uveal melanoma: a pilot study. Invest Ophthalmol Vis Sci.

[49] Reiniger IW, Schaller UC, Haritoglou C, Hein R, Bosserhoff AK, Kampik A, Mueller AJ (2005). “Melanoma inhibitory activity” (MIA): a promising serological tumour marker in metastatic uveal melanoma. Graefes Arch Clin Exp Ophthalmol.

[50] Missotten GS, Tang NE, Korse CM, Hurks HM, de Wolff-Rouendaal D, Keunen JE, Jager MJ, Bonfrer JM (2003). Prognostic value of S-100-beta serum concentration in patients with uveal melanoma. Arch Ophthalmol.

[51] Suesskind D, Schatz A, Schnichels S, Coupland SE, Lake SL, Wissinger B, Bartz-Schmidt KU, Henke-Fahle S (2012). GDF-15: a novel serum marker for metastases in uveal melanoma patients. Graefes Arch Clin Exp Ophthalmol.

[52] Bande MF, Santiago M, Blanco MJ, Mera P, Capeans C, Rodriguez-Alvarez MX, Pardo M, Pineiro A (2012). Serum DJ-1/PARK 7 is a potential biomarker of choroidal nevi transformation. Invest Ophthalmol Vis Sci.

[53] Bande MF, Santiago M, Mera P, Piulats JM, Blanco MJ, Rodríguez-Álvarez MX, Capeans C, Piñeiro A, Pardo M (2015). ME20-S as a Potential Biomarker for the Evaluation of Uveal MelanomaME20-S as a Potential Biomarker to Evaluate UM. Investigative Ophthalmology & Visual Science.

[54] Barisione G, Fabbi M, Gino A, Queirolo P, Orgiano L, Spano L, Picasso V, Pfeffer U, Mosci C, Jager MJ, Ferrini S, Gangemi R (2015). Potential Role of Soluble c-Met as a New Candidate Biomarker of Metastatic Uveal Melanoma.

[55] Frenkel S, Zloto O, Pe'er J, Barak V (2013). Insulin-like growth factor-1 as a predictive biomarker for metastatic uveal melanoma in humans. Invest Ophthalmol Vis Sci.

[56] Benassi MS, Pazzaglia L, Novello C, Quattrini I, Pollino S, Magagnoli G, Picci P, Conti A (2015). Tissue and serum IGFBP7 protein as biomarker in high-grade soft tissue sarcoma. Am J Cancer Res.

[57] Naumnik W, Ossolinska M, Plonska I, Chyczewska E, Niklinski J (2015). Circulating Thrombospondin-2 and FGF-2 in Patients with Advanced Non-small Cell Lung Cancer: Correlation with Survival. Adv Exp Med Biol.

