# Supplementary material for: In-depth proteomic profiling of the uveal melanoma secretome
Source: Oncotarget. 2016 Jul 6;7(31):49623–35. doi: 10.18632/oncotarget.10418 (PMC5226534; doi:10.18632/oncotarget.10418)
Supplement: Supplementary file 1 [file oncotarget-07-49623-s001.pdf]

In-depth proteomic profiling of the uveal melanoma secretome

Supplementary Materials

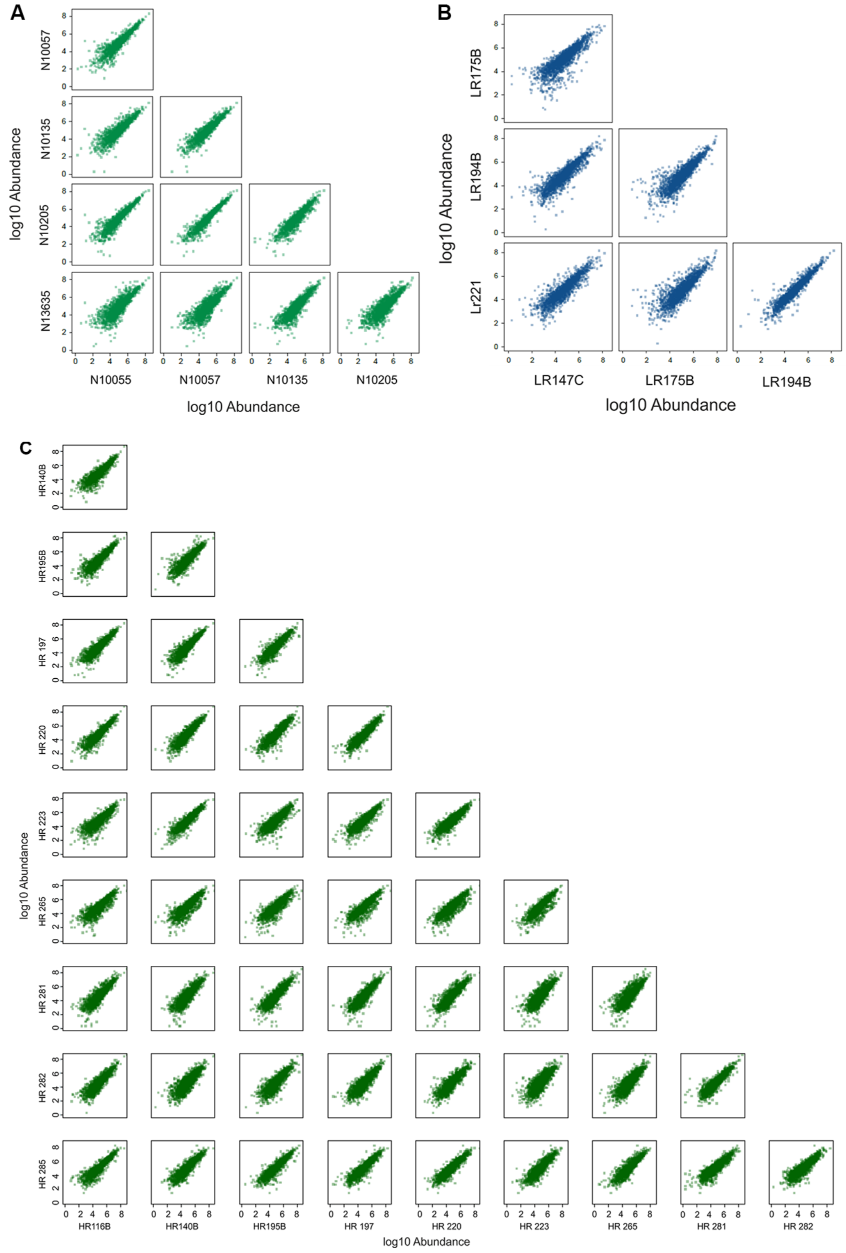

Supplementary Figure S1: Replicability of proteome analyses. For replicate secretome samples, the label-free quantitative profile of each sample was compared to the other samples from the same group and plotted as log transformed scatterplots. Data from NCM, LR-UM and HR-UM are shown in (**A**), (**B**) and (**C**) respectively.

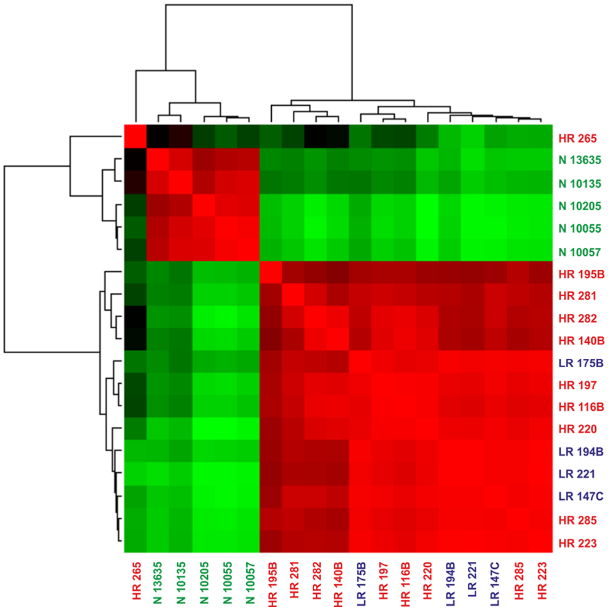

Supplementary Figure S2: Correlation heatmap of replicability of proteome analyses. For each set of secretome replicates, the Pearson correlation coefficient was high, and this was true also between LR and HR UM subsets. Between sample groups, however, the correlation coefficients were lower.

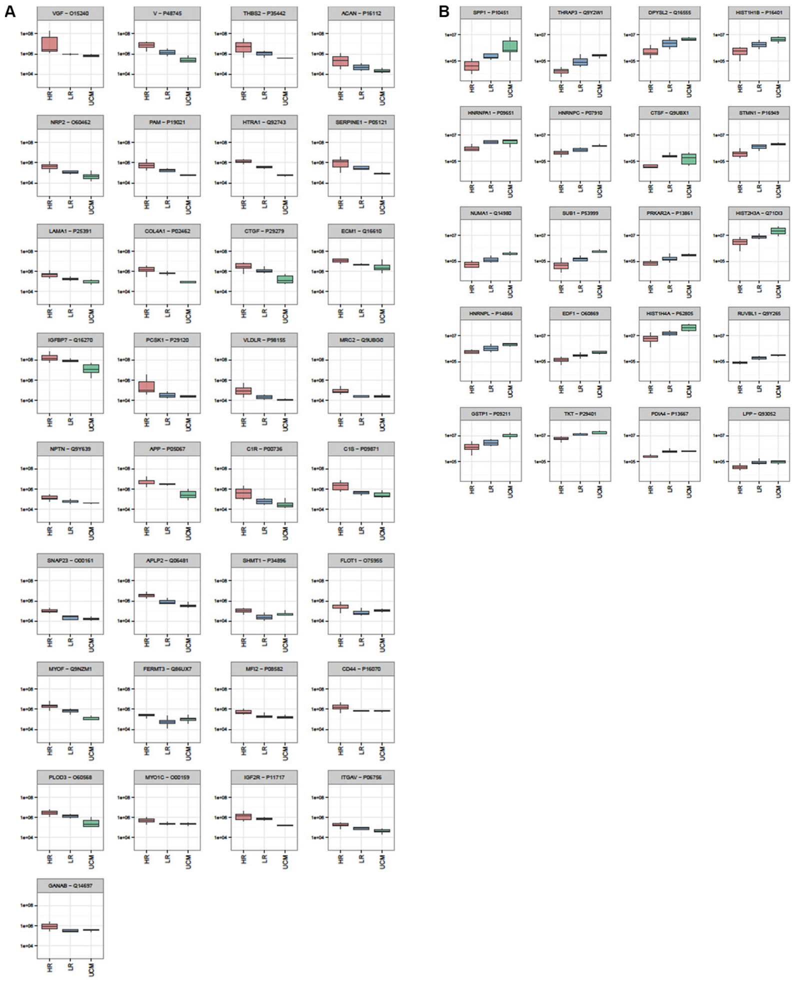

Supplementary Figure S3: Subset of proteins differentially expressed in HR-UM only compared with both LR-UM and NCM. Box and whisker plots of the 33 proteins up-regulated (**A**) or the 20 proteins that are down-regulated (**B**), between HR-UM and LR-UM/NCMs (see main manuscript for discussion).

Supplementary Table S1: Clinical, histopathological and genetic characteristics of the patient tumours used for secretome production

Sample No.	Age of patient at diagnosis (years)	Sex	LBD (mm)	UH (mm)	CBI	EOE	Cell type	Loops	Mitotic count/ 40 HPF	Chr3 status	Mets risk
S116-12	59	F	15.7	5.2	N	N	SPIN	Y	7	L	HR[¥]
S140-12	58	M	17.6	11	N	Y	MIX	N	5	L	HR*
S147-12	61	M	16.3	8.6	N	N	MIX	Y	5	N	LR*
S175-12	64	F	17.4	6.5	N	N	SPIN	N	3	N	LR
S194-12	41	M	12.7	11.6	N	N	MIX	N	3	N	LR
S195-12	77	F	14.7	5.7	N	N	EPI	N	7	L	HR*
S197-12	71	F	13.6	8	N	N	MIX	Y	6	L	HR
S220-12	80	F	18.2	12.5	N	Y	MIX	N	1	L	HR
S221-12	51	M	16.2	14.8	Y	N	SPIN	Y	4	N	LR
S223-12	67	M	18.7	12.2	Y	N	MIX	N	2	L	HR*
S265-12	76	F	15.4	7.8	N	N	SPIN	N	7	L	HR
S281-12	71	M	13	5.9	N	Y	MIX	N	4	L	HR*
S282-12	65	M	19.2	16.8	Y	N	MIX	Y	1	L	HR
S285-12	77	F	15.2	8.9	N	N	MIX	N	4	L	HR

*LBD: largest basal diameter; UH: tumour thickness; F: Female; M: Male; CBI: ciliary body involvement; EOE: extraocular extension; cell type: spindle, mixed, epithelioid; HPF: high power field; HR: high risk; LR: low risk; Chromosome 3 status: N-normal, L-loss, G-gain; *indicates death from metastatic disease; indicates death from other causes.*

Supplementary Table S2: Clinical features of the donor eyes used for culture of NCM

Sample No.	Age	Sex	Cause of death	Other history
NCM1	63	F	Renal cancer	Thyroid disease, hypercholesterolemia, atherosclerosis
NCM2	39	M	Cardiac arrest	Nil
NCM3	52	M	Pancreatic cancer	Non-insulin dependent diabetes
NCM4	55	M	Cardiac arrest	Nil
NCM5	17	M	Motor vehicle accident	Nil

F: female; M: male.

Supplementary Table S3: Activated biological functions in UM cells as compared with NCM. See Supplementary File 1

Supplementary Table S4: Canonical signalling pathways associated with UM. See Supplementary File 2

Supplementary Table S5: 325 differentially expressed secreted proteins. See Supplementary File 3
